# Supplementary material for: Understanding the heterogeneity of alloreactive natural killer cell function in kidney transplantation
Source: bioRxiv. 2023 Sep 5:2023.09.01.555962. Preprint. [Version 1] doi: 10.1101/2023.09.01.555962 (PMC10508724; doi:10.1101/2023.09.01.555962)
Supplement: Supplement 1 [file media-1.pdf]

Supplementary Table 1A. Regression model of 2-year eGFR in CTOT01

	Estimate	P Val
Intercept	57.9776	1e-04*
Thymoglobulin	1.0779	0.8784
Induction	-2.0563	0.8366
**Living transplant**	**22.537**	**0.0024***
Delayed Graft Function	16.121	0.4734
Acute Rejection	-1.8902	0.807
HLA-A/B/C mismatch	-1.0399	0.5746
**%Ksp37+**	**-4.8639**	**0.0408***

Supplementary Table 1B. Regression model of 2-year eGFR in CTOT01

	Estimate	P Val
Intercept	60.0487	2e-04*
Thymoglobulin	1.1814	0.8665
Induction	-2.5122	0.8018
**Living transplant**	**21.8542**	**0.0041***
Delayed Graft Function	16.395	0.4649
Acute Rejection	-1.7256	0.823
HLA-A/B/DR mismatch	-1.3429	0.5145
**%Ksp37+**	**-4.8235**	**0.0424***

Supplementary Table 2A. Regression model of 5-year eGFR in CTOT01

	Estimate	P Val
Intercept	66.9669	1e-04*
Thymoglobulin	-4.8275	0.5345
Induction	-10.0663	0.3842
Living transplant	9.8193	0.2241
Delayed Graft Function	25.4289	0.3582
Acute rejection	4.7733	0.5894
HLA-A/B/C mismatch	-0.4782	0.821
**%Ksp37+**	**-5.992**	**0.0229***

Supplementary Table 2B. Regression model of 5-year eGFR in CTOT01

	Estimate	P Val
Intercept	69.9433	1e-04*
Thymoglobulin	-4.8539	0.5306
Induction	-10.4846	0.3657
Living transplant	9.032	0.273
Delayed Graft Function	26.3281	0.3401
Acute rejection	4.8964	0.5792
HLA-A/B/DR mismatch	-1.0378	0.6293
**%Ksp37+**	**-5.961**	**0.0232***

Supplementary Table 3A. Regression model of 6-month eGFR in CTOT19

	Estimate	P Val
Intercept	54.749	0*
Delayed Graft Function	-0.443	0.9648
**%Ksp37+**	**-1.825**	**0.0321***

Supplementary Table 3B. Regression model of 6-month eGFR in CTOT19

	Estimate	P Val
Intercept	64.2575	0*
HLA-A/B/C mismatch	-2.8126	0.1524
%Ksp37+	-1.533	0.0639

Supplementary Table 3C. Regression model of 6-month eGFR in CTOT19

	Estimate	P Val
Intercept	55.7924	0*
Acute Rejection	-5.4084	0.7523
**%Ksp37+**	**-2.0835**	**0.0308***

Supplementary Table 4A. Regression model of 2-year eGFR in CTOT19

	Estimate	P Val
Intercept	64.5813	0*
Delayed Graft Function	-3.6054	0.7628
**%Ksp37+**	**-2.011**	**0.0454***

Supplementary Table 4B. Regression model of 2-year eGFR in CTOT19

	Estimate	P Val
Intercept	70.9803	0*
HLA-A/B/C mismatch	-2.1225	0.3716
%Ksp37+	-1.7936	0.0761

Supplementary Table 4C. Regression model of 2-year eGFR in CTOT19

	Estimate	P Val
Intercept	64.0183	0*
Acute Rejection	-8.7277	0.6709
%Ksp37+	-2.13	0.0611

Supplementary Table 5. Class I HLA alleles and KIR ligands in CTOT01

	HLA-A			HLA-B				HLA-C		
	Allele 1	Allele 2	Ligands	Allele 1	Allele 2	Ligands	-21 Dimorphism	Allele 1	Allele 2	Ligands
Donor #1	02:02	30:01	NA	07:02	53:01	Bw4/Bw6	MT	04:01	07:02	C1/C2
Recipient #1	02:02	30:01	NA	42:01	53:01	Bw4/Bw6	MT	04:01	17:01	C2/C2
Donor #2	01:01	32:01	Bw4	08:01	40:01	Bw6/Bw6	MT	02:02	07:01	C1/C2
Recipient #2	02:01	03:01	A03	08:01	38:01	Bw4/Bw6	MM	07:01	12:03	C1/C1
Donor #3	01:01	24:02	Bw4	08:01	35:03	Bw6/Bw6	MT	04:01	07:01	C1/C2
Recipient #3	01:01	24:02	Bw4	08:01	35:03	Bw6/Bw6	MT	04:01	07:01	C1/C2
Donor #4	02:01	02:01	NA	40:02	44:02	Bw4/Bw6	TT	02:02	05:01	C2/C2
Recipient #4	01:01	02:01	NA	40:02	57:01	Bw4/Bw6	TT	02:02	06:02	C2/C2
Donor #5	30:01	74:01	NA	08:01	44:03	Bw4/Bw6	MT	07:01	14:03	C1/C1
Recipient #5	32:01	74:01	Bw4	08:01	81:01	Bw6/Bw6	MM	07:01	08:04	C1/C1
Donor #6	01:01	24:02	Bw4	07:05	35:02	Bw6/Bw6	MT	04:01	15:05	C2/C2
Recipient #6	23:01	24:02	Bw4/Bw4	27:05	44:27	Bw4/Bw4	TT	01:02	07:04	C1/C1
Donor #7	68:01	68:01	NA	15:15	35:01	Bw6/Bw6	TT	01:02	07:02	C1/C1
Recipient #7	01:03	24:02	Bw4	15:01	73:01	Bw6/Bw6	MT	03:04	15:05	C1/C2
Donor #8	01:01	32:01	Bw4	08:01	14:01	Bw6/Bw6	MM	07:01	08:02	C1/C1
Recipient #8	02:01	03:01	A03	35:01	51:01	Bw4/Bw6	TT	04:01	15:02	C2/C2
Donor #9	02:01	32:01	Bw4	07:02	14:01	Bw6/Bw6	MM	07:02	08:02	C1/C1
Recipient #9	02:01	02:01	NA	07:02	08:01	Bw6/Bw6	MM	07:01	07:02	C1/C1
Donor #10	02:05	31:01	NA	35:08	41:01	Bw6/Bw6	TT	04:01	07:01	C1/C2
Recipient #10	23:01	66:01	Bw4	08:01	58:02	Bw4/Bw6	MT	03:04	06:02	C1/C2
Donor #11	01:01	03:01	A03	07:02	08:01	Bw6/Bw6	MM	07:01	07:02	C1/C1
Recipient #11	01:01	03:01	A03	07:02	08:01	Bw6/Bw6	MM	07:01	07:02	C1/C1
Donor #12	30:02	33:03	NA	15:16	57:03	Bw4/Bw4	TT	07:01	14:02	C1/C1
Recipient #12	34:02	36:01	NA	35:01	53:01	Bw4/Bw6	TT	04:01	04:01	C2/C2
Donor #13	23:01	33:03	Bw4	15:16	53:01	Bw4/Bw4	TT	06:02	14:02	C1/C2
Recipient #13	23:01	30:02	Bw4	53:01	58:02	Bw4/Bw4	TT	06:02	06:02	C2/C2
Donor #14	01:01	24:02	Bw4	07:02	08:01	Bw6/Bw6	MM	07:01	07:02	C1/C1
Recipient #14	01:01	03:01	A03	08:01	15:18	Bw6/Bw6	MT	07:01	07:04	C1/C1
Donor #15	02:01	66:01	NA	39:01	58:02	Bw4/Bw6	MT	06:02	07:02	C1/C2
Recipient #15	02:01	33:03	NA	35:01	53:01	Bw4/Bw6	TT	04:01	16:01	C1/C2
Donor #16	02:01	32:01	Bw4	14:01	44:02	Bw4/Bw6	MT	05:01	08:02	C1/C2
Recipient #16	02:01	03:01	A03	40:01	40:02	Bw6/Bw6	TT	02:02	03:04	C1/C2
Donor #17	26:01	32:01	Bw4	41:02	44:02	Bw4/Bw6	TT	05:01	17:03	C2/C2
Recipient #17	26:01	32:01	Bw4	08:01	44:02	Bw4/Bw6	MT	05:01	07:02	C1/C2
Donor #18	02:01	03:01	A03	07:02	40:02	Bw6/Bw6	MT	02:02	07:02	C1/C2
Recipient #18	02:01	25:01	NA	18:01	27:02	Bw4/Bw6	TT	02:02	12:03	C1/C2
Donor #19	02:01	24:02	Bw4	07:02	44:02	Bw4/Bw6	MT	05:01	07:02	C1/C2
Recipient #19	01:01	24:02	Bw4	27:05	44:02	Bw4/Bw4	TT	02:02	05:01	C2/C2
Donor #20	02:01	24:02	Bw4	40:01	40:02	Bw6/Bw6	TT	03:04	03:04	C1/C1
Recipient #20	02:01	03:01	A03	40:01	49:01	Bw4/Bw6	TT	03:04	07:01	C1/C1
Donor #21	02:01	30:02	NA	13:02	18:01	Bw4/Bw6	TT	05:01	06:02	C2/C2
Recipient #21	30:02	68:01	NA	08:01	18:01	Bw6/Bw6	MT	05:01	07:01	C1/C2
Donor #22	24:02	31:01	Bw4	51:01	51:01	Bw4/Bw4	TT	05:01	14:02	C1/C2
Recipient #22	02:01	03:01	A03	07:02	15:01	Bw6/Bw6	MT	04:01	07:02	C1/C2
Donor #23	02:01	24:02	Bw4	44:02	51:01	Bw4/Bw4	TT	05:01	16:02	C2/C2

Recipient #23	01:01	24:02	Bw4	08:01	51:01	Bw4/Bw6	MT	07:01	16:02	C1/C2
Donor #24	01:01	31:01	NA	51:01	52:01	Bw4/Bw4	TT	02:02	12:02	C1/C2
Recipient #24	01:01	02:01	NA	44:02	52:01	Bw4/Bw4	TT	05:01	12:02	C1/C2
Donor #25	01:01	01:01	NA	08:01	15:01	Bw6/Bw6	MT	03:04	07:01	C1/C1
Recipient #25	01:01	03:01	A03	08:01	53:01	Bw4/Bw6	MT	04:01	07:01	C1/C2
Donor #26	24:02	32:01	Bw4/Bw4	40:01	44:02	Bw4/Bw6	TT	03:04	05:01	C1/C2
Recipient #26	24:02	25:01	Bw4	39:01	44:02	Bw4/Bw6	MT	05:01	12:03	C1/C2
Donor #27	01:01	03:01	A03	07:02	08:01	Bw6/Bw6	MM	07:01	07:02	C1/C1
Recipient #27	02:NA	24:02	Bw4	52:NA	44:02	Bw4	TT	05:01	07:02	C1/C2
Donor #28	30:01	30:01	NA	42:01	42:01	Bw6/Bw6	MM	17:01	17:01	C2/C2
Recipient #28	03:01	33:03	A03	07:02	53:01	Bw4/Bw6	MT	04:01	07:02	C1/C2
Donor #29	02:01	02:01	NA	14:01	44:02	Bw4/Bw6	MT	05:01	08:02	C1/C2
Recipient #29	29:02	36:01	NA	45:01	53:01	Bw4/Bw6	TT	04:01	16:01	C1/C2
Donor #30	30:01	30:02	NA	42:01	58:01	Bw4/Bw6	MT	07:01	17:01	C1/C2
Recipient #30	02:01	30:02	NA	07:02	58:01	Bw4/Bw6	MT	07:01	07:02	C1/C1
Donor #31	01:01	32:01	Bw4	08:01	55:01	Bw6/Bw6	MT	05:01	07:01	C1/C2
Recipient #31	01:01	02:01	NA	08:01	15:03	Bw6/Bw6	MT	02:10	07:01	C1/C2
Donor #32	02:01	32:01	Bw4	15:01	44:02	Bw4/Bw6	TT	03:03	05:01	C1/C2
Recipient #32	02:01	32:01	Bw4	15:01	44:02	Bw4/Bw6	TT	03:03	05:01	C1/C2
Donor #33	02:01	02:01	NA	08:01	40:01	Bw6/Bw6	MT	03:04	07:01	C1/C1
Recipient #33	02:01	29:02	NA	40:01	44:03	Bw4/Bw6	TT	03:04	16:01	C1/C1
Donor #34	02:01	31:01	NA	44:02	51:01	Bw4/Bw4	TT	05:01	15:02	C2/C2
Recipient #34	02:01	31:01	NA	48:01	51:01	Bw4/Bw6	MT	08:01	15:02	C1/C2
Donor #35	02:01	68:01	NA	35:01	44:02	Bw4/Bw6	TT	05:01	15:02	C2/C2
Recipient #35	29:02	68:01	NA	35:01	44:03	Bw4/Bw6	TT	15:02	16:01	C1/C2
Donor #36	02:01	32:01	Bw4	15:01	39:01	Bw6/Bw6	MT	03:04	07:02	C1/C1
Recipient #36	01:01	32:01	Bw4	07:02	39:01	Bw6/Bw6	MM	07:02	07:02	C1/C1
Donor #37	01:01	11:01	A11	49:01	51:01	Bw4/Bw4	TT	07:01	15:02	C1/C2
Recipient #37	02:01	03:01	A03	07:02	15:01	Bw6/Bw6	MT	03:04	07:02	C1/C1
Donor #38	02:01	02:04	NA	40:01	51:01	Bw4/Bw6	TT	03:04	15:02	C1/C2
Recipient #38	24:02	24:02	Bw4/Bw4	07:02	44:03	Bw4/Bw6	MT	04:01	07:02	C1/C2
Donor #39	03:01	29:02	A03	14:02	44:03	Bw4/Bw6	MT	08:02	16:01	C1/C1
Recipient #39	02:01	24:02	Bw4	40:01	45:01	Bw6/Bw6	TT	03:04	16:01	C1/C1
Donor #40	01:01	11:01	A11	08:01	56:01	Bw6/Bw6	MT	01:02	07:01	C1/C1
Recipient #40	02:01	29:01	NA	18:01	57:01	Bw4/Bw6	TT	06:02	07:01	C1/C2
Donor #41	01:01	32:01	Bw4	13:02	40:02	Bw4/Bw6	TT	02:02	06:02	C2/C2
Recipient #41	01:01	02:01	NA	08:01	15:01	Bw6/Bw6	MT	03:03	07:01	C1/C1
Donor #42	23:01	34:02	Bw4	15:03	52:01	Bw4/Bw6	TT	02:10	16:01	C1/C2
Recipient #42	11:01	29:02	A11	07:02	07:02	Bw6/Bw6	MM	07:02	07:02	C1/C1
Donor #43	34:02	74:01	NA	44:03	57:03	Bw4/Bw4	TT	04:01	07:01	C1/C2
Recipient #43	02:02	34:02	NA	15:16	44:03	Bw4/Bw4	TT	04:01	14:02	C1/C2
Donor #44	01:01	23:17	Bw4	08:01	15:03	Bw6/Bw6	MT	02:10	07:02	C1/C2
Recipient #44	25:01	30:01	NA	07:02	53:01	Bw4/Bw6	MT	04:01	07:02	C1/C2
Donor #45	03:01	68:02	A03	15:10	15:10	Bw6/Bw6	TT	03:04	03:04	C1/C1
Recipient #45	23:01	68:02	Bw4	07:02	15:10	Bw6/Bw6	MT	03:04	15:05	C1/C2
Donor #46	02:03	26:01	NA	39:09	52:01	Bw4/Bw6	MT	07:02	07:02	C1/C1
Recipient #46	68:02	68:02	NA	15:10	35:01	Bw6/Bw6	TT	03:04	04:01	C1/C2
Donor #47	03:01	29:02	A03	07:02	44:03	Bw4/Bw6	MT	07:02	16:01	C1/C1

Recipient #47	03:01	29:02	A03	07:02	44:03	Bw4/Bw6	MT		04:01	07:02	C1/C2
Donor #48	02:01	03:01	A03	14:02	51:01	Bw4/Bw6	MT		08:02	12:03	C1/C1
Recipient #48	03:01	26:01	A03	15:01	38:01	Bw4/Bw6	MT		03:03	12:03	C1/C1
Donor #49	02:01	03:01	A03	14:02	51:01	Bw4/Bw6	MT		08:02	12:03	C1/C1
Recipient #49	11:01	23:01	A11/Bw4	57:01	58:01	Bw4/Bw4	TT		06:02	07:18	C1/C2
Donor #50	03:01	68:02	A03	14:02	57:01	Bw4/Bw6	MT		06:02	08:02	C1/C2
Recipient #50	02:01	11:01	A11	18:01	35:01	Bw6/Bw6	TT		04:01	07:01	C1/C2
Donor #51	33:03	68:01	NA	51:01	58:02	Bw4/Bw4	TT		06:02	16:01	C1/C2
Recipient #51	29:02	66:01	NA	44:03	58:02	Bw4/Bw4	TT		04:01	06:02	C2/C2
Donor #52	23:01	32:01	Bw4/Bw4	13:02	50:01	Bw4/Bw6	TT		06:02	06:02	C2/C2
Recipient #52	02:01	23:01	Bw4	27:05	50:01	Bw4/Bw6	TT		01:02	06:02	C1/C2
Donor #53	01:01	25:01	NA	08:01	58:01	Bw4/Bw6	MT		07:01	07:18	C1/C1
Recipient #53	01:01	25:01	NA	08:01	58:01	Bw4/Bw6	MT		07:01	07:18	C1/C1
Donor #54	02:01	24:02	Bw4	15:01	44:02	Bw4/Bw6	TT		03:03	05:01	C1/C2
Recipient #54	29:02	66:01	NA	47:03	53:01	Bw4/Bw6	TT		04:01	07:01	C1/C2
Donor #55	02:01	68:01	NA	35:01	35:08	Bw6/Bw6	TT		02:02	04:01	C2/C2
Recipient #55	30:01	74:01	NA	35:01	53:01	Bw4/Bw6	TT		04:01	04:01	C2/C2
Donor #56	01:01	02:01	NA	08:01	44:02	Bw4/Bw6	MT		05:01	07:01	C1/C2
Recipient #56	03:01	11:01	A03/A11	35:01	53:01	Bw4/Bw6	TT		04:01	04:01	C2/C2
Donor #57	30:02	68:01	NA	49:01	51:01	Bw4/Bw4	TT		07:01	15:02	C1/C2
Recipient #57	02:01	29:02	NA	07:06	35:01	Bw6/Bw6	MT		04:01	07:02	C1/C2
Donor #58	02:01	02:01	NA	15:01	18:01	Bw6/Bw6	TT		03:04	12:03	C1/C1
Recipient #58	02:01	33:03	NA	07:02	53:01	Bw4/Bw6	MT		04:01	07:02	C1/C2
Donor #59	02:05	23:01	Bw4	44:03	50:01	Bw4/Bw6	TT		04:01	06:02	C2/C2
Recipient #59	03:01	23:01	A03/Bw4	07:02	44:03	Bw4/Bw6	MT		04:01	07:02	C1/C2
Donor #60	02:01	23:01	Bw4	49:01	51:01	Bw4/Bw4	TT		03:03	07:01	C1/C1
Recipient #60	02:01	26:01	NA	38:01	55:01	Bw4/Bw6	MT		03:03	12:03	C1/C1
Donor #61	01:01	03:01	A03	35:01	57:01	Bw4/Bw6	TT		04:01	06:02	C2/C2
Recipient #61	01:01	03:01	A03	35:01	57:01	Bw4/Bw6	TT		04:01	06:02	C2/C2
Donor #62	24:02	32:01	Bw4/Bw4	35:01	57:01	Bw4/Bw6	TT		03:03	06:02	C1/C2
Recipient #62	03:01	23:01	A03/Bw4	15:03	42:01	Bw6/Bw6	MT		02:10	17:01	C2/C2
Donor #63	11:01	33:03	A11	07:02	18:01	Bw6/Bw6	MT		05:01	15:05	C2/C2
Recipient #63	11:01	32:01	A11/Bw4	08:01	18:01	Bw6/Bw6	MT		05:01	07:01	C1/C2
Donor #64	36:01	68:01	NA	52:01	53:01	Bw4/Bw4	TT		04:01	16:01	C1/C2
Recipient #64	02:02	68:01	NA	15:03	52:01	Bw4/Bw6	TT		02:10	16:01	C1/C2
Donor #65	02:01	68:02	NA	14:02	44:02	Bw4/Bw6	MT		05:09	08:02	C1/C2
Recipient #65	02:01	30:01	NA	15:03	40:02	Bw6/Bw6	TT		02:10	15:02	C2/C2
Donor #66	68:01	68:02	NA	45:01	49:01	Bw4/Bw6	TT		06:02	07:01	C1/C2
Recipient #66	02:01	24:02	Bw4	35:01	35:12	Bw6/Bw6	TT		04:01	04:01	C2/C2
Donor #67	01:01	74:01	NA	08:01	35:01	Bw6/Bw6	MT		07:01	07:01	C1/C1
Recipient #67	01:01	33:03	NA	18:01	57:03	Bw4/Bw6	TT		04:01	07:01	C1/C2
Donor #68	02:05	23:01	Bw4	07:02	35:01	Bw6/Bw6	MT		04:01	07:01	C1/C2
Recipient #68	23:01	30:02	Bw4	15:10	35:01	Bw6/Bw6	TT		03:04	07:01	C1/C1
Donor #69	03:01	30:02	A03	14:03	42:02	Bw6/Bw6	MM		08:02	17:01	C1/C2
Recipient #69	30:02	33:03	NA	42:02	58:01	Bw4/Bw6	MT		03:02	17:01	C1/C2
Donor #70	03:01	74:01	A03	07:02	35:01	Bw6/Bw6	MT		07:01	07:02	C1/C1
Recipient #70	02:01	03:01	A03	35:01	45:01	Bw6/Bw6	TT		07:01	16:01	C1/C1

Supplementary Table 6. Class I HLA allele and KIR ligands in CTOT19

	HLA-A			HLA-B				HLA-C		
ID	Allele 1	Allele 2	Ligands	Allele 1	Allele 2	Ligands	-21 Dimorphism	Allele 1	Allele 2	Ligands
Donor #1	02:01	25:01	NA	55:01	58:01	Bw4/Bw6	TT	03:03	07:18	C1/C1
Recipient #1	74:01	74:01	NA	27:05	35:01	Bw4/Bw6	TT	02:10	04:01	C2/C2
Donor #2	01:01	03:01	A03	35:01	44:02	Bw4/Bw6	TT	04:01	05:01	C2/C2
Recipient #2	03:01	68:01	A03	07:02	44:02	Bw4/Bw6	MT	05:01	07:02	C1/C2
Donor #3	01:01	02:01	NA	15:01	50:02	Bw6/Bw6	TT	03:03	06:02	C1/C2
Recipient #3	01:01	02:01	NA	15:01	50:02	Bw6/Bw6	TT	03:03	06:02	C1/C2
Donor #4	26:01	30:02	NA	07:02	51:01	Bw4/Bw6	MT	07:02	14:02	C1/C1
Recipient #4	03:01	30:02	A03	07:02	57:03	Bw4/Bw6	MT	07:02	07:18	C1/C1
Donor #5	02:01	24:02	Bw4	07:02	15:01	Bw6/Bw6	MT	03:04	07:02	C1/C1
Recipient #5	02:01	24:02	Bw4	07:02	15:01	Bw6/Bw6	MT	03:04	07:02	C1/C1
Donor #6	24:02	30:01	Bw4	13:02	39:06	Bw4/Bw6	MT	06:02	07:02	C1/C2
Recipient #6	30:01	33:03	NA	42:01	53:01	Bw4/Bw6	MT	04:01	17:01	C2/C2
Donor #7	02:01	68:02	NA	15:10	44:02	Bw4/Bw6	TT	03:04	05:01	C1/C2
Recipient #7	02:01	68:01	NA	15:18	44:02	Bw4/Bw6	TT	05:01	07:04	C1/C2
Donor #8	24:02	30:01	Bw4	18:01	42:01	Bw6/Bw6	MT	07:01	17:01	C1/C2
Recipient #8	30:01	30:01	NA	15:03	42:01	Bw6/Bw6	MT	02:10	17:01	C2/C2
Donor #9	02:01	26:01	NA	18:01	38:01	Bw4/Bw6	MT	07:01	12:03	C1/C1
Recipient #9	03:01	68:02	A03	42:01	44:02	Bw4/Bw6	MT	07:04	17:01	C1/C2
Donor #10	01:01	26:01	NA	27:05	44:03	Bw4/Bw4	TT	01:02	04:01	C1/C2
Recipient #10	01:01	26:01	NA	27:05	44:03	Bw4/Bw4	TT	01:02	04:01	C1/C2
Donor #11	02:01	02:01	NA	44:02	51:01	Bw4/Bw4	TT	05:01	15:02	C2/C2
Recipient #11	02:01	24:02	Bw4	15:01	35:01	Bw6/Bw6	TT	04:01	04:01	C2/C2
Donor #12	11:01	31:01	A11	40:02	44:03	Bw4/Bw6	TT	03:04	16:01	C1/C1
Recipient #12	02:01	66:01	NA	15:15	41:02	Bw6/Bw6	TT	01:02	17:03	C1/C2
Donor #13	03:01	68:01	A03	18:01	51:01	Bw4/Bw6	TT	05:01	15:02	C2/C2
Recipient #13	03:01	68:01	A03	18:01	51:01	Bw4/Bw6	TT	05:01	15:02	C2/C2
Donor #14	11:01	24:02	A11/Bw4	15:01	18:01	Bw6/Bw6	TT	03:03	12:03	C1/C1
Recipient #14	30:01	30:02	NA	15:03	42:01	Bw6/Bw6	MT	02:10	17:01	C2/C2
Donor #15	23:01	31:01	Bw4	44:03	55:01	Bw4/Bw6	TT	03:03	04:09	C1/C2
Recipient #15	01:01	03:01	A03	07:02	40:01	Bw6/Bw6	MT	03:04	07:02	C1/C1
Donor #16	23:01	31:01	Bw4	44:03	55:01	Bw4/Bw6	TT	03:03	04:09	C1/C2
Recipient #16	01:01	02:01	NA	08:01	49:01	Bw4/Bw6	MT	07:01	07:01	C1/C1
Donor #17	24:02	29:02	Bw4	13:02	55:01	Bw4/Bw6	TT	01:02	06:02	C1/C2
Recipient #17	02:01	23:01	Bw4	07:02	41:01	Bw6/Bw6	MT	07:01	07:02	C1/C1
Donor #18	01:01	32:01	Bw4	15:01	51:01	Bw4/Bw6	TT	03:03	14:02	C1/C1
Recipient #18	11:01	31:01	A11	44:02	51:01	Bw4/Bw4	TT	05:01	15:02	C2/C2
Donor #19	02:01	11:01	A11	51:01	56:01	Bw4/Bw6	TT	01:02	02:02	C1/C2
Recipient #19	23:17	68:01	Bw4	08:01	39:05	Bw6/Bw6	MM	07:01	07:02	C1/C1
Donor #20	11:01	24:02	A11/Bw4	13:02	35:01	Bw4/Bw6	TT	04:01	06:02	C2/C2
Recipient #20	03:01	03:01	A03/A03	35:01	45:01	Bw6/Bw6	TT	04:01	05:01	C2/C2
Donor #21	01:01	24:02	Bw4	07:02	35:01	Bw6/Bw6	MT	04:01	07:02	C1/C2
Recipient #21	02:02	30:01	NA	42:02	53:01	Bw4/Bw6	MT	04:01	17:01	C2/C2

Donor #22	02:01	11:01	A11	15:01	51:01	Bw4/Bw6	TT	03:03	15:02	C1/C2
Recipient #22	02:01	03:01	A03	07:02	51:01	Bw4/Bw6	MT	07:02	14:02	C1/C1
Donor #23	24:02	26:01	Bw4	38:01	48:01	Bw4/Bw6	MM	08:03	12:03	C1/C1
Recipient #23	02:01	33:03	NA	45:01	58:01	Bw4/Bw6	TT	03:02	16:01	C1/C1
Donor #24	03:01	30:02	A03	15:03	35:01	Bw6/Bw6	TT	02:10	04:01	C2/C2
Recipient #24	66:01	68:02	NA	35:01	41:02	Bw6/Bw6	TT	16:01	17:03	C1/C2
Donor #25	02:01	68:03	NA	39:05	52:01	Bw4/Bw6	MT	03:03	07:02	C1/C1
Recipient #25	24:02	26:01	Bw4	35:14	57:01	Bw4/Bw6	TT	04:01	06:02	C2/C2
Donor #26	02:01	02:01	NA	44:02	45:01	Bw4/Bw6	TT	05:01	06:02	C2/C2
Recipient #26	02:01	02:01	NA	44:02	45:01	Bw4/Bw6	TT	05:01	06:02	C2/C2

Supplementary Table 7. Class I HLA allele and KIR ligands in healthy cohort

	HLA-A			HLA-B				HLA-C		
ID	Allele 1	Allele 2	Ligands	Allele 1	Allele 2	Ligands	-21 Dimorphism	Allele 1	Allele 2	Ligands
Healthy #1	31:01	32:01	Bw4	15:01	40:01	Bw6/Bw6	TT	03:03	03:04	C1/C1
Healthy #2	24:02	30:02	Bw4	18:01	35:01	Bw6/Bw6	TT	04:01	06:02	C2/C2
Healthy #3	23:01	31:01	Bw4	38:01	49:01	Bw4/Bw4	MT	07:01	12:03	C1/C1
Healthy #4	02:01	11:01	A11	51:01	52:01	Bw4/Bw4	TT	12:02	15:02	C1/C2
Healthy #5	24:02	66:01	Bw4	38:01	52:01	Bw4/Bw4	MT	12:02	12:03	C1/C1
Healthy #6	24:02	33:03	Bw4	13:01	53:01	Bw4/Bw4	TT	03:04	04:01	C1/C2
Healthy #7	25:01	31:01	NA	18:01	35:12	Bw6/Bw6	TT	04:01	12:03	C1/C2
Healthy #8	02:05	26:01	NA	41:01	52:01	Bw4/Bw6	TT	07:01	12:02	C1/C1
Healthy #9	23:01	24:02	Bw4/Bw4	18:01	18:01	Bw6/Bw6	TT	07:01	07:04	C1/C1
Healthy #10	02:01	03:01	A03	35:01	51:01	Bw4/Bw6	TT	04:01	15:02	C2/C2
Healthy #11	02:01	30:04	NA	44:02	49:01	Bw4/Bw4	TT	05:01	07:01	C1/C2
Healthy #12	32:01	33:01	Bw4	08:01	14:02	Bw6/Bw6	MM	07:01	08:02	C1/C1
Healthy #13	24:02	26:01	Bw4	18:01	38:01	Bw4/Bw6	MT	12:03	12:03	C1/C1
Healthy #14	01:01	02:01	NA	08:01	57:01	Bw4/Bw6	MT	06:02	07:01	C1/C2
Healthy #15	30:02	68:01	NA	15:03	53:01	Bw4/Bw6	TT	02:10	04:01	C2/C2
Healthy #16	02:01	26:01	NA	08:01	53:01	Bw4/Bw6	MT	03:04	04:01	C1/C2
Healthy #17	11:01	24:02	A11/Bw4	15:01	15:25	Bw6/Bw6	TT	03:03	07:26	C1/C1
Healthy #18	02:01	03:01	A03	51:01	53:01	Bw4/Bw4	TT	01:02	16:01	C1/C1
Healthy #19	01:01	02:03	NA	40:06	57:01	Bw4/Bw6	TT	06:02	15:02	C2/C2
Healthy #20	01:01	26:01	NA	44:02	53:01	Bw4/Bw4	TT	04:01	05:01	C2/C2

Supplementary Table 8. Flow cytometry panel for profiling stimulator cell ligands

	Antigen	Vendor	Catalog #	Clone
Panel 1	HLA-A/B/C	BioLegend	311418	W6/32
	MICA/B	BioLegend	320912	6D4
	CD86	BioLegend	374214	BU63
	HLA-F	BioLegend	373208	3D11/HLA-F
	CD112	Miltenyi	130-122-770	REA1195
Panel 2	CD80	BioLegend	305225	2D10
	HLA-G	BioLegend	335912	87G
	ULBP1	R&D Systems	FAB1380A	170818
	CD54	BioLegend	353117	HA58
	HLA-C	BD Biosciences	747594	DT-9
Panel 3	CD155	BioLegend	337634	SKII.4
	HLA-E	BioLegend	342604	3D12
	PD-L1	BioLegend	329736	29E.2A3
	ULBP2/5/6	R&D Systems	FAB1298G	165903
	CD58	BioLegend	330918	TS2/9
Viability	Zombie NIR	BioLegend	423105	

Supplementary Table 9. CyTOF Panel

Metal Conjugate	Antigen	Vendor	Catalog #	Clone	Function
89Y	CD45	Fluidigm	3089003B	HI30	Lineage marker; leukocyte
111Cd	LILRB1	BioLegend	333702	GHI/75	NK cell
112Cd	CD8	BioLegend	301002	RPA-T8	Lineage marker; T cell
113Cd	CD38	Miltenyi	130-122-307	REA572	NK cell, B cell
114Cd	CD3	BioLegend	300402	UCHT1	Lineage marker; T cell
115In	CD14	BioLegend	301802 & 301843	M5E2	Lineage marker; myeloid & other
115In	CD19	BioLegend	302202	HIB19	Lineage marker; B cell
115In	CD33	BioLegend	303402	WM53	Lineage marker; myeloid & other
116Cd	CD57	BioLegend	359602	HNK-1	NK and T cell terminal maturation
141Pr	TCRvd2	BioLegend	331402	B6	T cell
142Nd	CCL4	Miltenyi	130-095-212	REA511	NK cell effector function
143Nd	XCL1	R&D Sys	AF695	109001	NK cell effector function
144Nd	Ksp37	BioLegend	custom	TDA3	NK cell effector function
145Nd	NKG2D	Miltenyi	130-122-332	REA797	NK cell
146Nd	DNAM-1	Miltenyi	130-092-479 & 130-126-485	DX11 & REA1040	NK cell
147Sm	PLZF	R&D Sys	MAB2944	6318100	Transcription factor
148Nd	KIR3DL1/L2	Miltenyi	130-126-489	REA970	NK cell education
149Sm	CD25	Fluidigm	3149010B	2A3	NK and T cell effector function
150Nd	TCF-1/7	BioLegend	655202	7F11A10	Transcription factor
151Eu	CCL5	R&D Sys	MAB278-100	21445	NK cell effector function
152Sm	KIR3DL1	Miltenyi	130-092-555	DX9	NK cell education
153Eu	TIM-3	Fluidigm	3153008B	F38-2E2	Exhaustion
154Sm	TIGIT	Fluidigm	3154016B	MBSA43	Exhaustion
155Gd	NKp46	Miltenyi	130-124-522	REA808	NK cell
156Gd	KIR2DL2/L3	Miltenyi	130-122-346	REA1006	NK cell education
158Gd	KIR2DL1	Miltenyi	130-122-279	REA284	NK cell education
159Tb	CD56	Miltenyi	130-108-016	REA196	NK cell
160Gd	NKG2A	Miltenyi	130-122-329	REA110	NK cell education
161Dy	Ki-67	Fluidigm	3161007B	B56	Proliferation
162Dy	CD27	Fluidigm	3162009B	L128	NK cell, T cell, B cell
163Dy	CXCR3	Fluidigm	3163004B	G025H7	NK and T cell effector function
164Dy	NKG2C	Miltenyi	130-122-278	REA205	NK cell
165Ho	KIR2DL3	Miltenyi	130-122-280	REA147	NK cell education
166Er	KIR2DL1/S1	Miltenyi	130-122-345	REA1010	NK cell education
167Er	FceR1g	EMD millipore	06-727	polyclonal	NK cell education
168Er	IFNg	Fluidigm	3168005B	B27	NK cell effector function
169Tm	Granzyme K	BioLegend	370502	GM26E7	NK cell effector function
170Er	CD122	Fluidigm	3170004B	Tu27	NK and T cell effector function
171Yb	Granzyme B	Fluidigm	3171002B	GB11	NK cell effector function
172Yb	CD107a	Miltenyi	130-124-536	REA792	NK cell effector function
173Yb	CD137	Fluidigm	3173015B	4B4-1	NK cell effector function
174Yb	TOX	Miltenyi	custom	REA473	Transcription factor
175Lu	Perforin	Fluidigm	3175004B	B-D48	NK cell effector function
176Yb	CD4	Fluidigm	3176010B	RPA-T4	T cell
198Pt	HLA-DR	Miltenyi	130-122-299	REA805	Lineage marker; myeloid & other
209Bi	CD16	Fluidigm	3209002B	3G8	NK cell

Supplementary Table 10. Flow cytometry panel for NK cell sorting and killing assay

Antigen	Vendor	Catalog #	Clone	Function
CD19	BioLegend	302244	HIB19	B cell
CD56	BioLegend	362550	5.1H11	NK cell
CD3	BioLegend	317346	OKT3	T cell
NKG2A	Miltenyi	130-128-163	REA110	NK cell education
NKG2C	Miltenyi	130-117-398	REA205	NK cell education
KIR3DL1/L2	Miltenyi	130-116-180	REA970	NK cell education
KIR2D	Miltenyi	130-117-483	REA1042	NK cell education
CD107a	Miltenyi	130-111-624	REA792	NK cell effector function
Ksp37	BioLegend	346603	TDA3	NK cell effector function
Zombie NIR	BioLegend	423106		Fixable viability dye
Propidium iodide	Life Technologies	P3566		Viability dye
