# Supplementary material for: Understanding the heterogeneity of alloreactive natural killer cell function in kidney transplantation
Source: bioRxiv. 2023 Sep 5:2023.09.01.555962. Preprint. [Version 1] doi: 10.1101/2023.09.01.555962 (PMC10508724; doi:10.1101/2023.09.01.555962)
Supplement: Supplement 2 [file media-2.pdf]

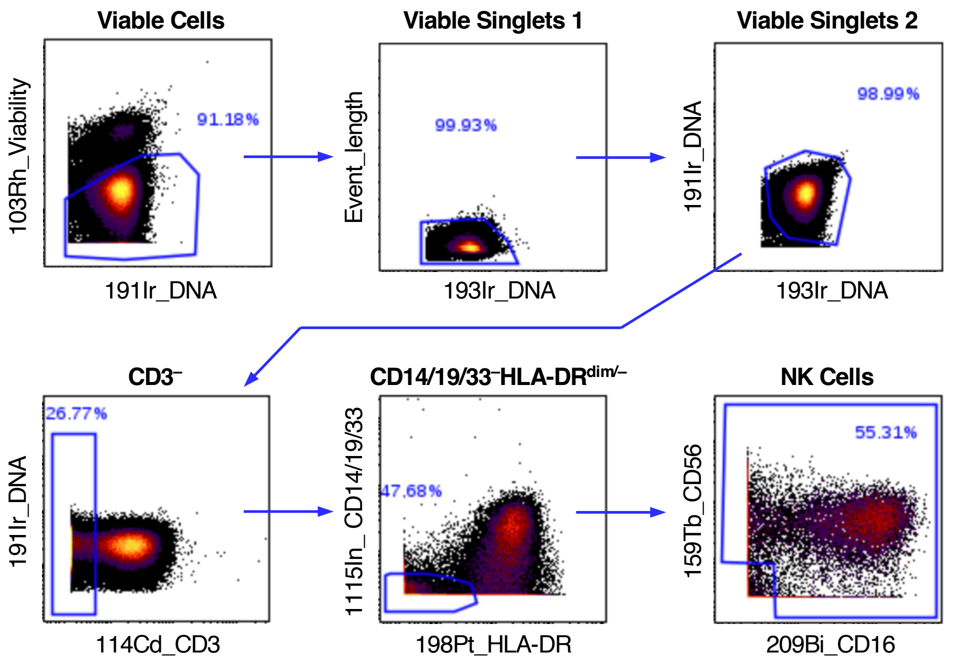

Supplementary Figure 1. Gating strategy for NK cells on CyTOF sample. Cryopreserved PBMC were recovered overnight in 10 ng/mL rhIL-15 and profiled by CyTOF following stimulation. Representative FACS plots show manual gating of NK cells prior to unsupervised clustering and downstream analyses.

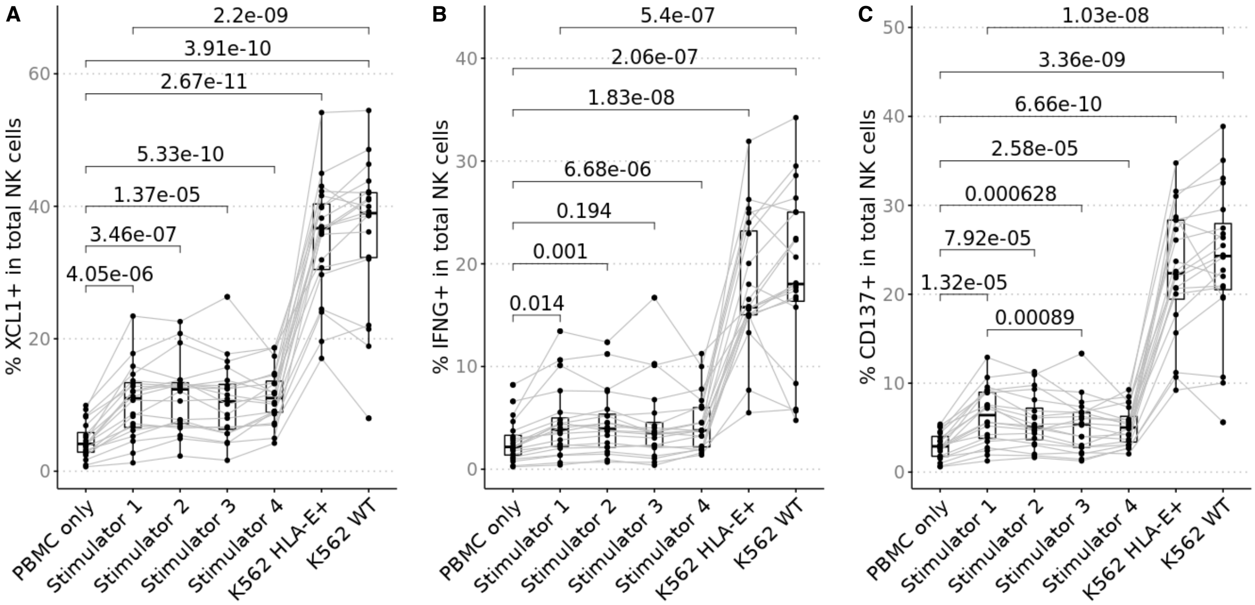

Supplementary Figure 2. Activation of alloreactive NK cells varies between healthy donors and stimulator cell. Cryopreserved PBMC from healthy donor (n=20) were recovered overnight in 10 ng/mL rhIL-15 and stimulated for 6 hours 3:1 E/T with 4 distinct allo-stimulator lines, K562 WT and K562 HLA-E+. Results were profiled by CyTOF. Percentage of (A) XCL1+, (B) IFNγ+, (C) CD137+ NK cells across healthy donors increases in response to stimulators. There were no differences between Stimulators 1-4 except for %CD137+ between Stimulator 1 and Stimulator 3. All Stimulators induced less activation than K562 WT and K562 HLA-E+, and there was no difference between K562 WT and K562 HLA-E+. *P* value was calculated using 2-sided paired Student's *t*-test and values were adjusted for multiple testing with Bonferroni correction.

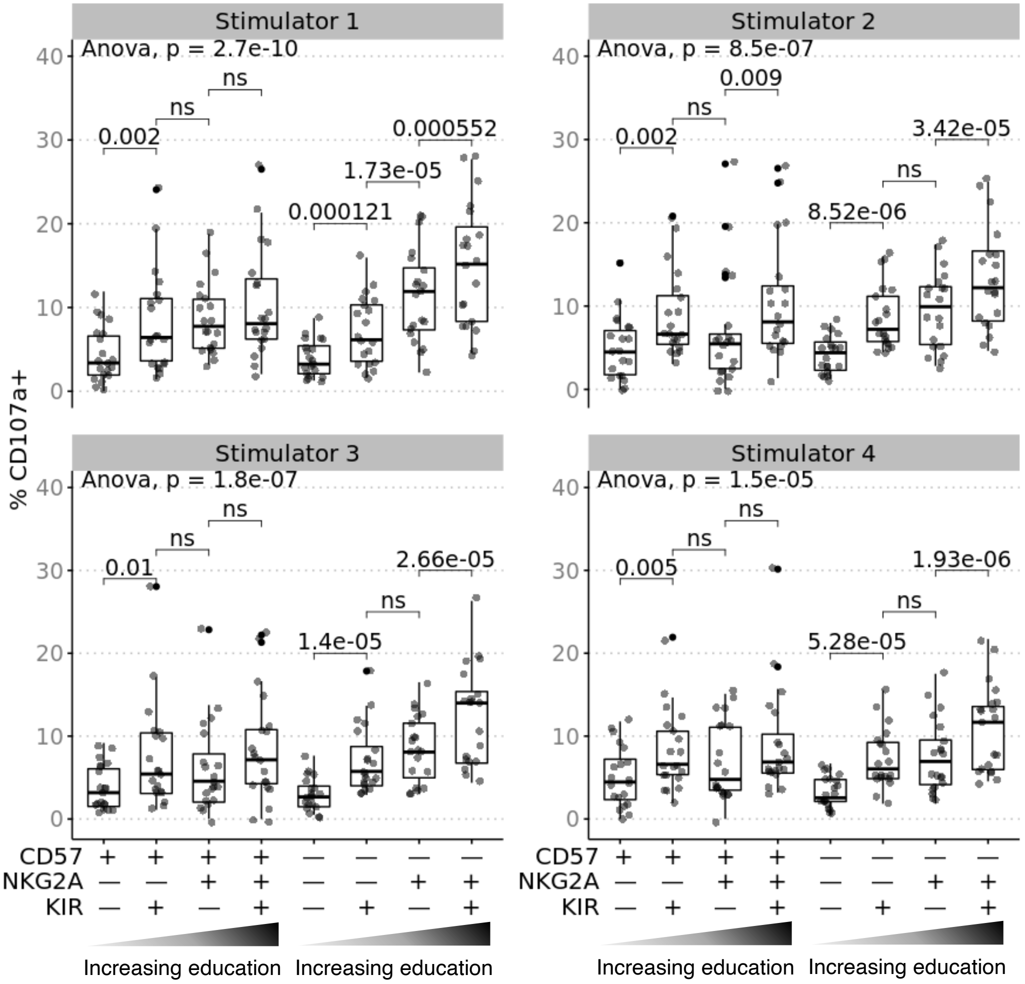

Supplementary Figure 3. Educated NK cell subsets are more responsive to missing-self when stimulated with all allo-stimulator cell lines. Cyropreserved PBMC from healthy donor (n=20) were recovered overnight in 10 ng/mL rhIL-15 and stimulated for 6 hours 3:1 E/T with 4 distinct allo-stimulator lines, K562 WT and K562 HLA-E+. Results were profiled by CyTOF. NK cell subsets were defined by gating on combinatorial expression of educating inhibitory receptors, NKG2A, KIR3DL1, KIR3DL2, KIR2DL1/S1, KIR2DL2 and KIR2DL3 and the CD57 maturation marker. NKG2A-expressing and KIR-expressing subsets produce more CD107a in response to B cell allo-stimulators 1-4. *P* value was calculated using 2-sided paired Student's *t*-test and values were adjusted for multiple testing with Bonferroni correction; ns indicates $p > 0.1$.

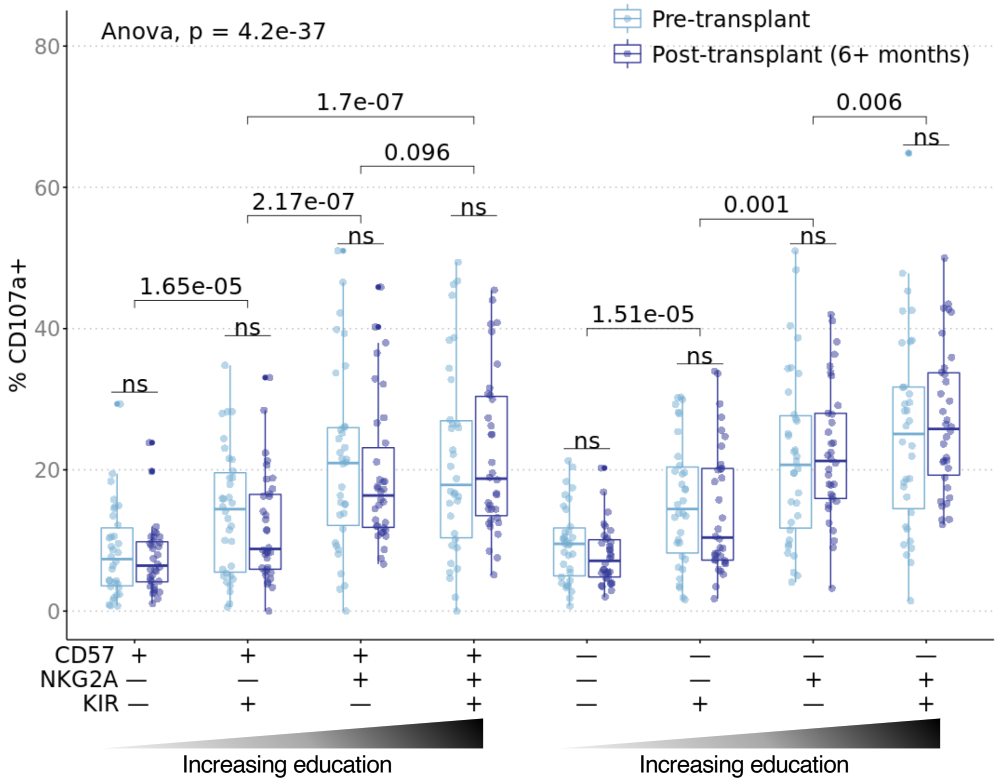

Supplementary Figure 4. Alloreactivity of educated NK cells transcend donor differences and is maintained post-transplant. Cryopreserved pre- and post-transplant PBMC from CTOT01 kidney transplant (n=70) were recovered overnight in 10 ng/mL rhIL-15 and stimulated with donor allo-stimulator B cells for 6 hours at 3:1 E/T. Results were profiled by CyTOF and subsets were defined by gating on $CD56^{dim}$ NK cells followed by combinatorial expression of educating inhibitory receptors, NKG2A, KIR3DL1, KIR3DL2, KIR2DL1 and KIR2DL3 and the CD57 maturation marker. Uneducated NK cells, defined as KIR^{+} NK cells in recipients without HLA that encode cognate ligand, were removed from the total KIR^{+} population of that recipient. The increase in alloreactivity of the educated subsets compared to uneducated subsets in the pre- and post-transplant timepoints are consistent with the results in Figure 5. *P* value was calculated as one-way ANOVA as indicated and as 2-sided paired Student's *t*-test; *t*-test *p* values were adjusted for multiple testing with Bonferroni correction and ns indicates $p > 0.1$.

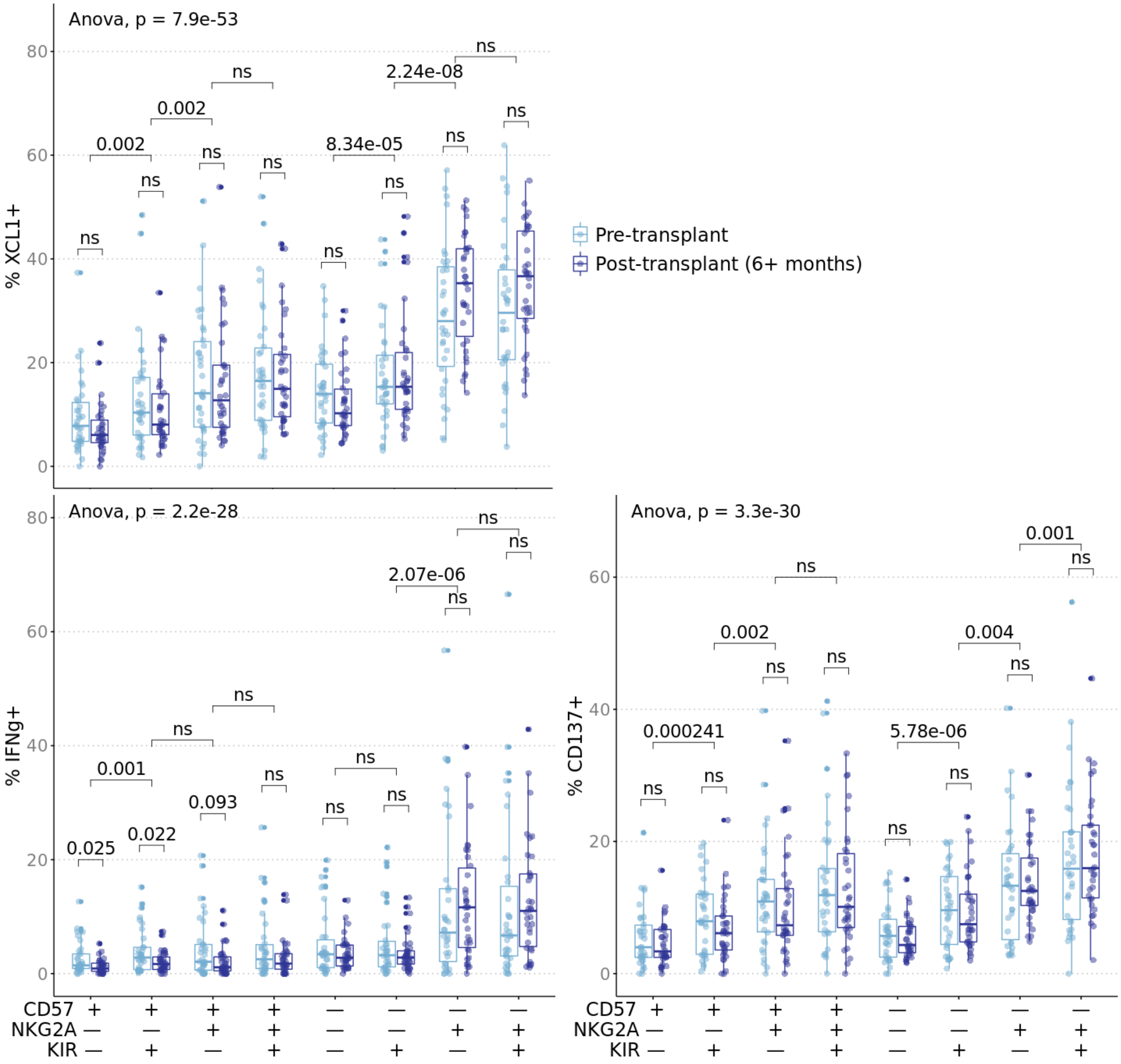

Supplementary Figure 5. Educated NK cells maintain alloreactivity post-transplant. Pre- and post-transplant PBMC from CTOT01 kidney transplant (n=70) were recovered overnight in 10 ng/mL rhIL-15 and stimulated with donor allo-stimulator B cells for 6 hours at 3:1 E/T. Results were profiled by CyTOF and subsets were defined by gating on $CD56^{dim}$ NK cells followed by combinatorial expression of educating inhibitory receptors, NKG2A, KIR3DL1, KIR3DL2, KIR2DL1 and KIR2DL3 and the CD57 maturation marker. Upon stimulation with donor cells, recipient $NKG2A^{+}KIR^{+}$ subsets produced the most XCL1, IFNγ, and CD137 while the uneducated $NKG2A^{-}KIR^{-}$ subsets produces the least XCL1, IFNγ, and CD137. The effect of NKG2A/KIR education on CD137 and CD107a expression in response to donor cells persisted 6+ months post-transplant (n=34). IFNγ production in $CD57^{+}NKG2A^{-}KIR^{-}$ and $CD57^{+}NKG2A^{-}KIR^{+}$ NK cells decreased post-transplant. *P* value was calculated as one-way ANOVA as indicated and as 2-sided paired Student's *t*-test. *t*-test *p* values were adjusted for multiple testing with Bonferroni correction and ns indicates $p > 0.1$.

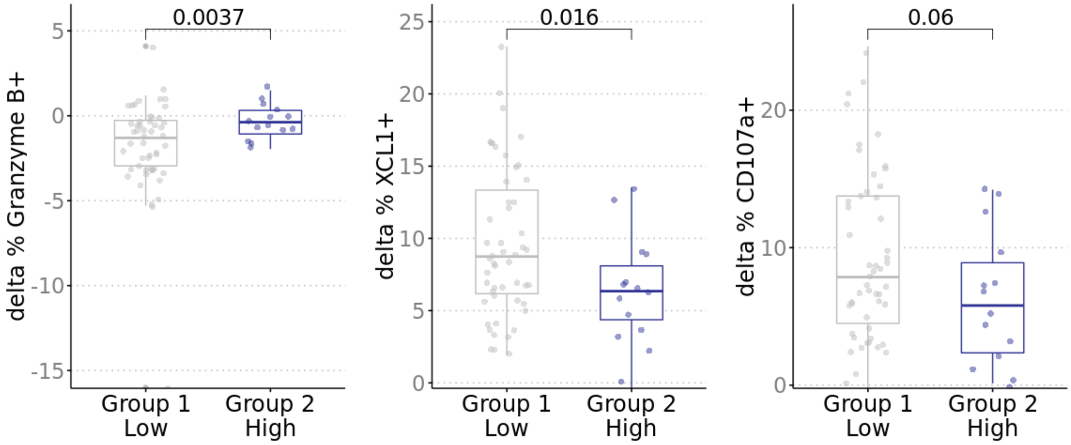

Supplementary Figure 6. High HLA-E expression on donor cells inhibit recipient NKG2A⁺NKG2C⁻ NK cells. Donor stimulator groups were defined by expression of HLA-E where 75th percentile was high (n=16) and remaining were low (n=51). Hierarchical clustering defined 14 donors with high HLA-E expression and similar expression of other inhibitory/activating NK ligands and 2 donors with high HLA-E expression that also expressed higher CD112/HLA-F expression compared to other high HLA-E donors. One donor expressed CD112 at 3.3 standard deviations greater than the mean and the other donor expressed HLA-F at 1.8 standard deviations from mean. These two donors were excluded from this analysis. Change in percent positive of CD107a, XCL1 and Granzyme B in NKG2A⁺NKG2C⁻ cells from baseline was greater when recipient cells were stimulated by donors with lower HLA-E. Nominal *p* value was calculated using 2-sided unpaired Student's *t*-test.

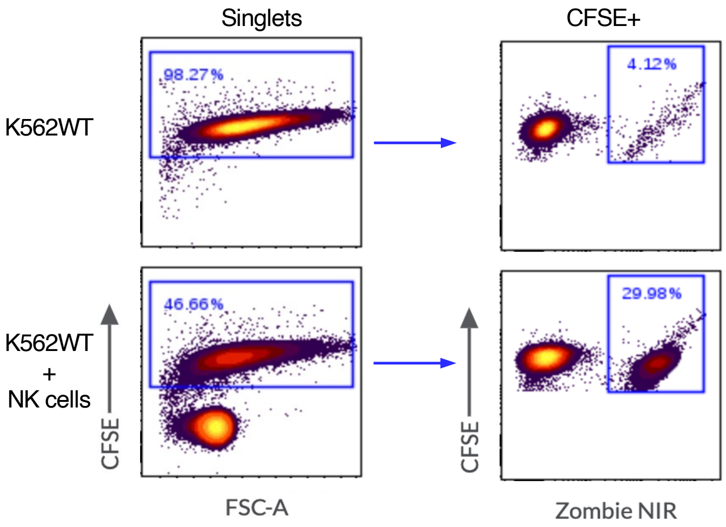

Supplementary Figure 7. Gating strategy for NK cell killing assays. PBMC from healthy donor buffy coats were isolated by Ficoll density centrifugation and rested overnight in 10 ng/mL IL-15. PBMCs were enriched for NK cells by negative selection and flow-sorted for NKG2A^{+}KIR^{+}NKG2C^{-}, NKG2A^{+}KIR^{-}NKG2C^{-}, NKG2A^{-}KIR^{+}NKG2C^{-}, and NKG2A^{-}KIR^{-}NKG2C^{-} subsets. NK cell subsets were stimulated with CFSE-stained K562 wildtype and allo-stimulator B cells. Representative FACS plots show gating on CFSE^{+} stimulator cells. Zombie NIR viability dye staining identifies stimulator cells that have died in co-culture with NK cells.
